# Spillover and spillback risks of ectoparasites by an invasive squirrel *Callosciurus erythraeus* in Kanto region of Japan

**DOI:** 10.1016/j.ijppaw.2022.07.006

**Published:** 2022-07-31

**Authors:** Hirotaka Katahira, Yuya Eguchi, Saki Hirose, Yukino Ohtani, Azusa Banzai, Yusaku Ohkubo, Tatsuki Shimamoto

**Affiliations:** a School of Life and Environmental Science, Azabu University, 1-17-71 Fuchinobe, Chuo-ku, Sagamihara, Kanagawa, 252-5201, Japan; b School of Veterinary Nursing and Technology, Nippon Veterinary and Life Science University, 1-7-1 Kyonancho, Musashino, Tokyo, 180-8602, Japan; c Center for Data Assimilation Research and Applications, Joint Support Center for Data Science Research, Research Organization of Information and Systems10-3 Midori-cho, Tachikawa, Tokyo, 190-8562, Japan; d The Institute of Statistical Mathematics, 10-3 Midori-cho, Tachikawa, Tokyo, 190-8562, Japan

**Keywords:** Pallas's squirrel, Tick, Chigger mite, Lice, Flea, Zoonosis, Management

## Abstract

Invasive organisms can alter host-parasite relationships in a given ecosystem by spreading exotic parasites and/or becoming a new reservoir for native ones. Since these problems affect management programs of the invasive host organisms, it is necessary to monitor them individually. The Pallas's squirrel *Callosciurus erythraeus* is an invasive arboreal mammal introduced into Japan that threatens to exacerbate ecological and public health problems by spreading native and exotic parasites. However, only limited surveys have been available especially for ectoparasites, using the traditional combing method in which the possibility of oversight is inherent. Here, we evaluated the ectoparasite occurrences in Kanto region of Japan, using the whole-shaving method as an alternative approach. As a result of examining 52 hosts from two invaded districts (Yokohama and Yokosuka), chigger mites (*Leptotrombidium* spp.) and fleas (*Ceratophyllus anisus* and *Ceratophyllus indages indages*) were newly recovered in addition to the previously reported tick (*Haemaphysalis flava*) and exotic lice (*Enderleinellus kumadai* and *Neophaematoponis callosciuri*). The parasite burdens were higher in Yokosuka and in male host individuals, affecting infracommunity richness and composition. Our findings on the variety of native and exotic ectoparasites, at higher abundances in some cases than previously known, may suggest that both the spillover and spillback risks need to be adjusted upwards.

## Highlights

• Ectoparasite infections were examined for Pallas's squirrels invaded in Kanto region of Japan.
• A variety of native and exotic ectoparasites with high abundance in some cases was found.
• Infracommunity composition changed depending on the invading environment and host features.
• Spillover and spillback risks may need to be adjusted upwards in this country.

## Graphical abstract

**Figure:**
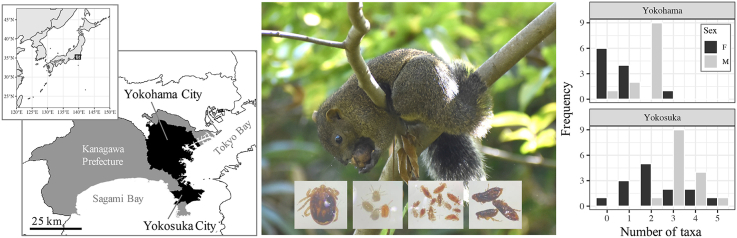

## 1 Introduction

Invasive organisms alter the relationships between host species and pathogens in a given ecosystem (Dunn, 2009; Dunn et al., 2012; Hatcher et al., 2012a, b). Exotic pathogens introduced along with invasive hosts sometimes switch hosts so that they cause severe damage to native organisms (Torchin et al., 2002; Torchin and Mitchell, 2004). This phenomenon is called pathogen pollution or the spillover effect (Lymbery et al., 2014). Simultaneously, invasive organisms can become additional host resources for native pathogens (e.g. Rauque et al., 2003; Mastisky and Veres, 2010), which break the balance between native organisms and pathogens by enhancing the infections, called the spillback effect (Kelly et al., 2009; Lymbery et al., 2014). Since the control and/or elimination of these pathogens affects management goals and control measures for the targeted host organisms (Rushton et al., 2000, 2006; see also Dunn and Hatcher, 2015), their trends should be taken into consideration.

The Pallas's squirrel *Callosciurus erythraeus* is an arboreal species originally distributed in southeastern Asia from the southeast of China to the east of India and Taiwan (Thorington et al., 2012), though it has been introduced world-wide into Argentina, France, Belgium, the Netherlands, and Hong Kong in addition to Japan (reviewed in Lurz et al., 2013); only Belgium has succeeded in eradicating this squirrel (Adriaens et al., 2015). In Japan, it was introduced as a companion animal for the first time in the early 20th century, but has invaded various areas from East to Southwest Japan due to escape and anthropogenic dispersion (Miyamoto et al., 2004; Oshida et al., 2007; Tamura, 2015). This species has the ability to flexibly adapt to various environments including urban green spaces and rural woods, causing economic losses involving crops, trees, and house damage (e.g. Hori et al., 2006; see also Tamura, 2015). Negative conflicts with the native Japanese squirrel *Sciurus lis* are also a concern as distribution expands (Miyamoto et al., 2004). Local governments are thus enforcing extermination programs, but exclusion has not been sufficiently achieved (Tamura, 2015).

In addition to other ecological and economic problems, both endo- and ecto-metazoan parasites have been reported from this invasive squirrel as are the cases in other countries (Gozzi et al., 2021). The endoparasitic threadworm *Strongyloides callosciureus* and two ectoparasitic sucking lice *Enderleinellus kumadai* and *Neohaematopinus callosciuri* are exotic parasite species co-introduced along with the host in Japan (Kaneko, 1954; Shinozaki et al., 2004a, b; Sato et al., 2007; Miyabe et al., 2016; Eguchi et al., 2022). Among native species, three ectoparasites, a mouse flea *Ceratophyllus anisus*, a tick *Haemaphysalis flava*, and a poultry red mite *Dermanyssus* cf. *gallinae* have been found from an established host population in Kamakura City and Zushi City, Kanagawa Prefecture of Kanto region, East Japan (Shinozaki et al., 2004a, b; Nakamura and Fukase, 2022). Unidentified endoparasitic nematode species belonging to the families Kathranidae and Heligmonelidae have also been found from this invasive squirrel, but require taxonomic examination to discriminate whether they are native or not (Wang, 1981; Matsudate et al., 2003; Miyabe et al., 2016).

Ectoparasites are especially known as vectors that carry zoonotic pathogens infecting humans (Gratz, 1999; Diaz, 2006). For example, fleas can transmit bacterial diseases, such as plague and rickettsiosis (Azad et al., 1990; see also Bitam et al., 2010), while ticks harbor pathogens that cause Japanese spotted fever, severe fever with thrombocytopenia syndrome (SFTS), and rickettsiosis (e.g. Yamaji et al., 2018; Kinoshita et al., 2021; see also Dantas-Torres et al., 2012). Although the squirrel invasion could be a matter of public health, there has been limited investigation of these ectoparasites in Japan (Shinozaki et al., 2004a, b). Considering that parasite infection is affected by available host resources (e.g. Patterson and Ruckstuhl, 2013), it is expected that ectoparasites, both native and exotic, have become too numerous as the number of established host individuals has increased in recent years (Miyamoto et al., 2004; see also Tamura, 2015).

We therefore investigated the abundance and diversity of ectoparasites on Pallas's squirrels to determine their current infection status in two invaded districts of Japan. To evaluate the exact infection status, we employed the fur shaving method, instead of the combing method that is commonly used in ectoparasite examinations. Although the combing method is simple and quick, it carries a risk of underestimation due to its lack of precision (e.g. Hopkins, 1949).

## 2 Materials and methods

### 2.1 Study area

**Fig. 1:**
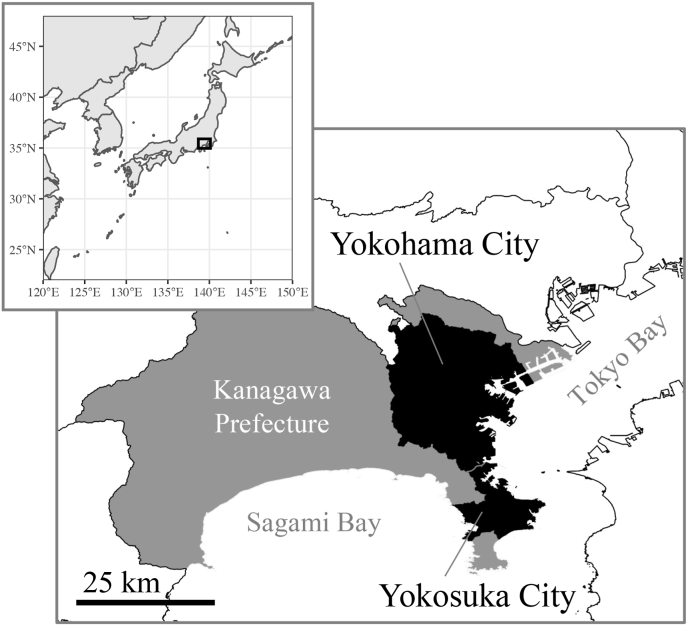
Location map of the study areas. The Pallas's squirrels examined in this study were collected by extermination programs operated by the local governments of Yokohama and Yokosuka. Detailed information on the sampling localities is refrained due to the intension of the cooperative organizations.

The host squirrels were captured from unspecified number of green spaces in Yokosuka and Yokohama districts of Kanagawa Prefecture in Kanto region (Fig. 1), during the term from November 2018 to May 2020. In these areas, local governments operate extermination programs for Pallas's squirrels and hire a contractor to collect the squirrels. The squirrel specimens were indiscriminately captured under the programs and immediately frozen after euthanized by using carbon dioxide gas.

The euthanized individuals were immediately each packed in a plastic bag, kept in a freezer, and subsequently transferred to the laboratory of Nippon Veterinary and Science University and Azabu University, where biometric measurements such as head-body length (HBL) and body weight (BW) were taken after thawing, and internal organs were removed for other research (e.g. Eguchi et al., 2022). The remaining carcasses were used for the following examination.

### 2.2 Parasite sampling

Whole-body fur of each host individual was shaved using a razor on a plastic tray (ca. 445 mm, 325 mm, and 70 mm in length, width and depth) and temporarily stored in a plastic bag (ca. 140 mm and 10 mm in length and width). Waste pieces on the tray were filtered with a sieve with mesh size of 150 μm diameter (Sieve No. 150, Tokyo SANPO) and carefully flushed by distilled water so as not to overlook any parasite specimens. Residue on the mesh and the shaved fur was examined under a stereomicroscope (SZX7, Olympus). All ectoparasites found were collected using tweezers and stored in glass bottles filled with 70% ethanol.

For the species identification, parasite specimens were tentatively placed in 30% glycerol, sealed with nail polish under a cover glass, and observed using a light microscope (BX51, Olympus). Identification for each taxon followed previous descriptions (Acari: Kumada, 1965; Ehara, 1980; Yamaguti, 1981; Takada and Takahashi, 2019, Anoplura: Kim and Ludwig, 1978; Johnson, 1959; Kaneko, 1954, Siphonaptera: Sakaguti, 1962). Morphological identification of tick specimens was furthermore affirmed with the DNA barcode of the 16S rDNA region provided by Takano et al. (2014) following their protocol. Ticks and lice were classified by their developmental stages. The parasite prevalence and abundance were calculated using these categories according to the definitions of Bush et al. (1997).

### 2.3 Statistical analysis

To evaluate explanatory variables associated with parasite occurrences represented as infracommunity richness, generalized linear mixed models (GLMM) were fitted to the number of parasite taxa found on an individual host. In this analysis, a Poisson distribution was assumed as the distribution of the response variable (Goater et al., 1987) while considering individual host variance of the infracommunity richness as a random effect. The sampling area (Yokohama and Yokosuka), season (divided tentatively into two seasons, i.e. a cold season during October to March when chigger mites are known to increase and a hot season during April and September) (Tamiya, 1962), host body size (HBL), host body condition index (hereafter BCI, calculated as residual deviance from a regression of BW to HBL while considering the sampling areas and host sex; Schulte-Hostedde et al., 2005), and host sex were tested as candidate explanatory variables. The model selection was based on the lowest score of Bayesian information criterion (BIC).

The similarity of the parasite infracommunity observed among the host individual was then expressed as the Chao index with adding a dummy column to account for no infection cases (Chao et al., 2005). Based on this data, infracommunity composition with discriminating developmental stages of ticks and louse was visualized using non-metric multidimensional scaling (NMDS). The effect of probable variables, as is the case in the above GLMM analyses, on the composition of NMDS scores was analyzed using the envfit function with 10,000 permutations.

All the above analyses were performed in R 4.0.3 (R Development Core Team, 2020), with the glmmTMB package and vegan package.

## 3 Results

### 3.1 Host data

A total of 52 host individuals were examined. Of these, 12 males and 11 females were from Yokohama captured during August to October 2019, while 15 males and 14 females were from Yokosuka captured during December 2018 to May 2020 (Table 1). Host individuals from both districts represented similar ranges of HBL and BW, although the average BW values in males and females were higher in Yokohama. Significant differences in the variance of the host body conditions were not found between the sexes and sampling areas (p > 0.98 in a nested ANOVA).

**Table 1:** Host and parasite data.

							
			Yokohama		Yokosuka		
Host data							
		Sex	M	F	M	F	
		No. of examaind	12	11	15	14	
		Mean HBL (mm) (min-max)	231.08 (213–246)	235.82 (228–243)	232.13 (209–248)	231.07 (214–246)	
		Mean BW (g) (min-max)	356.67 (300–420)	370.00 (310–410)	306.67 (240–360)	310.71 (220–400)	
		Mean BCI (g) (min-max)	0.54 (-38.06–31.11)	0.82 (-43.05–33.71)	1.00 (-53.36–32.76)	0.76 (-44.30–43.07)	
Parasite infection							Origin
**Acari**							
	*Haemaphysalis flava*						Native
	Nymph	Prevalence (%)	0	0	2 (13.33)	4 (28.57)	
		No. of recovered	–	–	2	5	
		Mean abundance (max)	–	–	0.13 (1)	0.36 (2)	
	Larva	Prevalence (%)	0	0	2 (13.33)	1 (7.14)	
		No. of recovered	–	–	2	1	
		Mean abundance (max)	–	–	0.13 (1)	0.07 (1)	
	Total	Prevalence (%)	0	0	3 (20.00)	5 (35.71)	
		No. of recovered	–	–	4	6	
		Mean abundance (max)	–	–	0.27 (2)	0.43 (2)	
	*Leptotrombidium* spp.						Unidentified
	Larva	Prevalence (%)	0	0	6 (40.00)	4 (28.57)	
		No. of recovered	–	–	53	94	
		Mean abundance (max)	–	–	3.53 (22)	6.71 (91)	
**Anoplura**							
	*Enderleinellus kumadai*						Exotic*
	Adult	Prevalence (%)	9 (75.00)	2 (18.18)	11 (73.33)	2 (14.29)	
		No. of recovered	287	2	87	19	
		Mean abundance (max)	23.92 (172)	0.18 (1)	5.80 (26)	1.36 (14)	
	Larva	Prevalence (%)	4 (33.33)	0	10 (66.67)	2 (14.29)	
		No. of recovered	135	–	49	3	
		Mean abundance (max)	11.25 (106)	–	3.27 (27)	0.21 (2)	
	Total	Prevalence (%)	9 (75.00)	2 (18.18)	12 (80.00)	2 (14.29)	
		No. of recovered	422	2	136	22	
		Mean abundance (max)	35.17 (278)	0.18 (1)	9.07 (53)	1.57 (16)	
	*Neohaematopinus callosciuri*						Exotic
	Adult	Prevalence (%)	9 (75.00)	2 (18.18)	14 (93.33)	8 (57.14)	
		No. of recovered	111	5	149	105	
		Mean abundance (max)	9.25 (43)	0.45 (3)	9.93 (29)	7.50 (40)	
	Larva	Prevalence (%)	11 (91.67)	4 (36.36)	15 (100.00)	11 (78.57)	
		No. of recovered	260	5	415	200	
		Mean abundance (max)	21.67 (80)	0.45 (2)	27.67 (92)	14.29 (52)	
	Total	Prevalence (%)	11 (91.67)	4 (36.36)	15 (100.00)	11 (78.57)	
		No. of recovered	371	10	564	305	
		Mean abundance (max)	30.92 (96)	0.91 (4)	37.60 (119)	21.79 (92)	
**Siphonaptera**							
	*Ceratophyllus anisus*						Native
	Adult	Prevalence (%)	0	1 (9.09)	8 (53.33)	5 (35.71)	
		No. of recovered	–	1	12	12	
		Mean abundance (max)	–	0.09 (1)	0.80 (3)	0.86 (4)	
	*Ceratophyllus indages indages*						Native
	Adult	Prevalence (%)	0	0	6 (40.00)	5 (35.71)	
		No. of recovered	–	–	16	15	
		Mean abundance (max)	–	–	1.07 (8)	1.07 (8)	
							

*This species is first described from an introduced specimen of the Pallas's squirrel in Japan (Kaneko, 1954), but its presence has subsequently been confirmed from the squirrels inhabiting in the native habitats of Thailand (Johnson, 1959).

### 3.2 Parasite infection

Six taxa were recovered from the examined hosts (Table 1). They were seven nymphs and three larvae of the tick *H. flava*, 147 larvae of chigger mites *Leptotropmbidium* spp., 395 adults and 187 larvae of the lice *E. kumadai*, 370 adults and 880 larvae of the louse *N. callosciuri*, 25 adults of the flea *C. anisus*, and 31 adults of the flea *Ceratophyllus indages indages.*

**Fig. 2:**
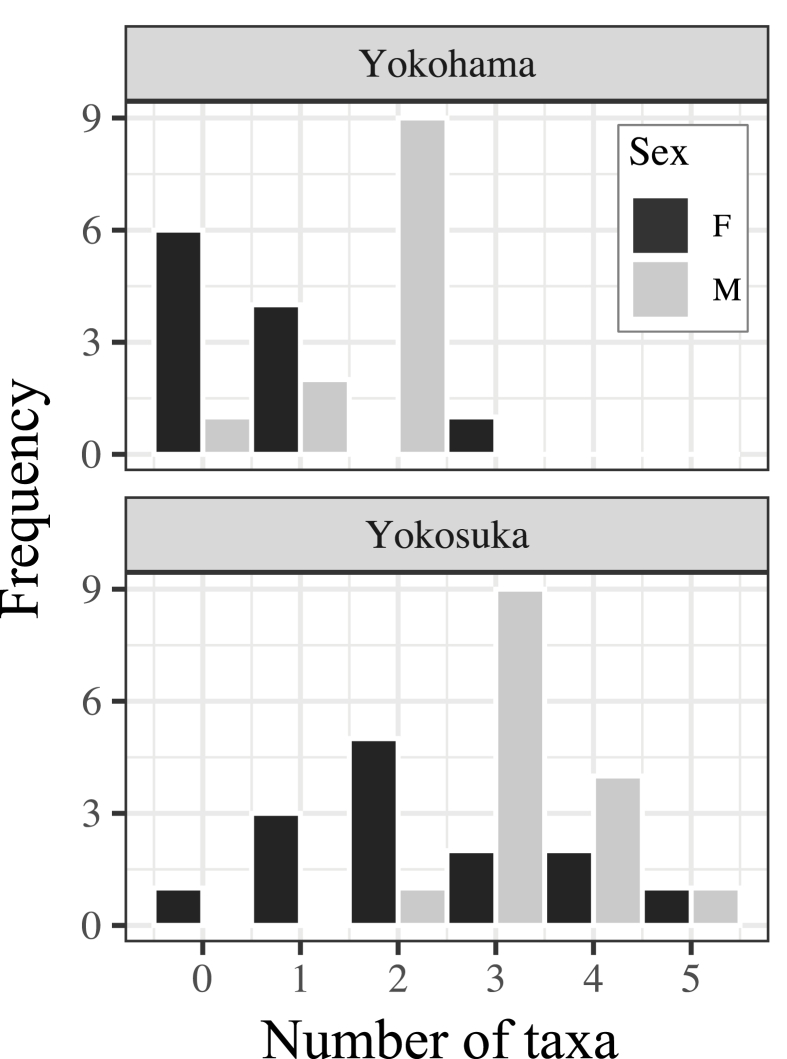
Infracommunity richness by discriminating the study area and host sex.

Two dominant sucking lice, *E. kumadai* and *N. callosciuri*, were at especially high prevalence and mean abundance in the host males in both areas (Table 1), with 278 and 119 individuals at the maximum abundance, respectively. The chigger mites, *Leptotrombidium* spp., were found to be the next most dominant parasites in Yokosuka, with 91 individuals at the maximum abundance. For the infracommunity richness, the number of host individuals infected with more than one parasite taxon was up to 44 cases (84.62%) in total (Fig. 2). The percentages by sampling area were 69.57% (91.67% in males and 45.45% in females) in Yokohama and 96.55% (100% in males and 92.86% in females) in Yokosuka.

As a result of GLMM fitting for the infracommunity richness, a model comprised of the sampling area and host sex presented the lowest BIC value (Supplementary Table S1). The model including the host body condition in addition to these explanatory variables was second, with a difference in the BIC value of 2.84 from the lowest model. In the selected lowest model, the expected number of taxa found in the host individuals from Yokosuka was 2.41 [ = exp (0.88)] times higher than that from Yokohama (standard error = 0.22, z-value = 3.97, p < 0.001, Fig. 2). Male hosts furthermore harbored 1.67 [ = exp (0.51)] times more parasite taxa than females (standard error = 0.20, z-value = 2.56, p = 0.011).

**Fig. 3:**
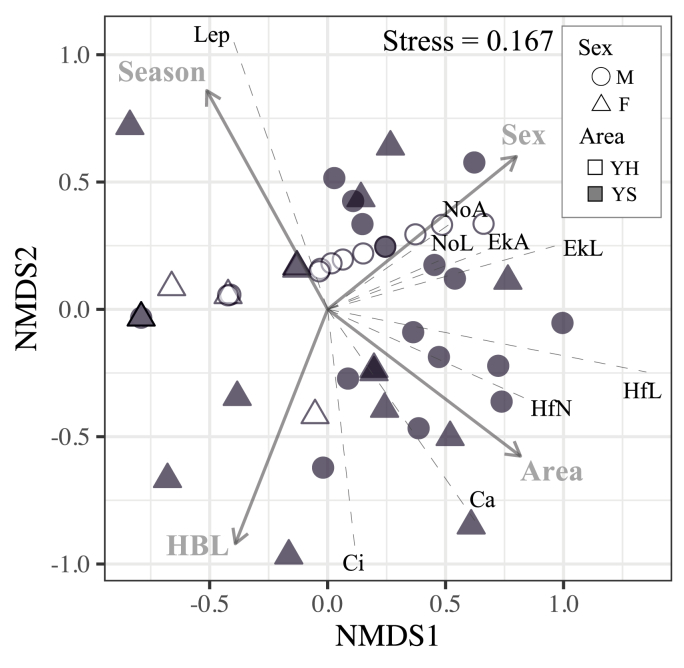
NMDS plot for the parasite infracommunity composition recovered from 52 host individuals. The influence of each parasite's abundance by discriminating the developmental stage on the score components of the two axes is represented by broken lines. The relationships with environmental variables are indicated by gray arrows. Abbreviations are as follows: M: male, F: female, YH: Yokohama, YS: Yokosuka, HfN: nymph of *Haemaphysalis flava*, HfL: larva of *H. flava*, Lep: larva of *Leptotrombidium* spp., EkA: adult of *Enderleinellus kumadai*, EkL: larva of *E. kumadai*, NoA: adult of *Neohaematopinus callosciuri*, NoL: larva of *N. callosciuri*, Ca: *Ceratophyllus anisus*, Ci: *Ceratophyllus indages indages*.

Infracommunity composition represented as two-dimensional NMDS scores was changed by the sampling area (p = 0.0011), season (p = 0.041), host body size (p = 0.049), and host sex (p < 0.001) (Fig. 3). The host body condition was not supported as the related variable with the infracommunity composition (p = 0.14).

## 4 Discussion

This study revealed that the Pallas's squirrel, which has invaded and established in Japan, harbors a variety of native and exotic ectoparasites with high abundances that in some cases are more than previously known. Past reports applying the combing method have recovered only three ectoparasite species (Shinozaki et al., 2004a, b); moreover, single species abundances were less than 10, though there are seasonal changes with a peak in February. The reason for the low parasite abundance being reported in earlier studies cannot be determined, but the combing method may only capture a small fraction of the ectoparasites present (e.g. Hopkins, 1949), or the environmental conditions of the study were such that the parasite abundances were low. Alternatively, the ectoparasite abundances were indeed low in the past. In any case, the present shaving approach, which detected a much higher number of parasite species and individuals, may suggest that both spillover and spillback risks of native and exotic ectoparasites need to be adjusted upwards. The present targeted mammal, the Pallas's squirrel, is still expanding its distribution in Japan (Tamura, 2015). Along with this expansion, local host-parasite relationships are likely to change throughout the country.

In the view of the parasite infracommunity, most host individuals are infected with at least one parasite taxon is a noteworthy finding. This suggests that host dispersal, even if it is only one individual, can result not only in the unintentional spread of the parasite into new areas but also affect the balance of local parasitism. In addition, infracommunity composition changes depending on the invading environment and host features. This may be due to differences in native mammal diversity as natural reservoirs for native ectoparasites, sexual differences in the home range size and overlap in the host squirrel (Tamura et al., 1989) and/or a simple increase in attachment area along with body size (e.g. Esser et al., 2016). Especially when considering male dispersal habits in this host species (Tamura et al., 1989), the spillover and spillback effects may be more significant in male squirrels than females.

The tick and fleas found in this study are all native species in Japan. These taxa are known to be zoonotic disease vectors. For example, severe fever with thrombocytopenia syndrome (SFTS) has recently become problematic as a tick-borne disease in Japan (Kobayashi et al., 2020). Members of the genus *Haemaphysalis*, including *H. flava*, are thought to be potential vectors of the viruses that cause SFTS (Ejiri et al., 2018; Kodama et al., 2021). SFTS is currently suspected to be associated with sika deer, whose numbers are recently increasing (Lundu et al., 2018; Saijo, 2018; Yamaji et al., 2018; see also Doi et al., 2020), but other wild mammals can also become important reservoirs of the vector ticks. Two exotic mammals, raccoons and palm civets, already established in the study area have been recently reported to potentially influence tick populations (Doi et al., 2018, 2021). As a third exotic mammal, the Pallas's squirrel, also deserves attention because it may increase/decrease the number of tick individuals as suggested in the present finding. It is thus conceivable that the combined effects of these exotic mammals could affect the risks of tick-borne diseases, including SFTS.

In the case of the mouse flea *C. anisus*, Pallas's squirrel may act as a novel resource that positively affects the parasite population size in addition to the original hosts such as house rats and other domestic mammals (Sakaguti, 1962). Congeners have been reported to have bacterial pathogens (Lipatova and Paulauskas, 2012; Aivelo and Tschirren, 2020), suggesting that the same can be expected for this species, and increasing population size near human habitats would not be favored from a public health view. The other flea, *C. indages indages*, has so far been limitedly reported from the Eurasian red squirrel *Sciurus vulgaris orientis*, which is distributed in Hokkaido of northern Japan (Sakaguti, 1962), but it is unclear why this flea is parasitizing Pallas's squirrel in Kanagawa Prefecture. Further research is required to determine whether this parasite is naturally distributed or has appeared for some anthropogenic reason.

Chigger mites, *Leptotrombidium* spp. could not be determined strictly as native or exotic because we used a simple method for specimen preparation. However, the native congeners have been known to transmit rickettsia and cause endemic tsutsugamushi disease in Japan (Tamiya, 1962; Yamaji et al., 2018; Yoshikura, 2018; Kinoshita et al., 2021). In Miura peninsula, where Yokosuka is located and the present study was conducted, cases have been reported in the past and the disease has been named Miura fever or Nobi fever (Shioya, 1959; Kadosaka, 2020). Although rodents and mice are thought to be the original reservoirs for the problematic chigger mites (Tamiya, 1962), there is a possibility that the presence of Pallas's squirrel affects their population. Incidentally, if the exotic host is not suitable for the harmful native parasites, it can be a dead-end host with a role of decreasing the parasite infection risk in the given ecosystem (known as dilution effect, Norman et al., 1999). In addition, the invasive host has a possibility to take a role of the pathogen reservoir causing a profound or moderate effect on transmission to other sympatric mammals and humans regardless of candidate vector abundance (see Matuschka and Spielman, 1992). Therefore, a comprehensive survey including other sympatric mammals is thus required to evaluate various effects including removal or amplification of pathogenic risk.

Both the lice, *E. kumadai* and *N. callosciuri*, are thought to be exotic species introduced into Japan along with the Pallas's squirrel (Kaneko, 1954; Shinozaki et al., 2004a). Although they are reported only from this squirrel, spillover to the native Japanese squirrel may occur as the invasive host's distributional range continues to expand and overlap the native's range. In Kanto region, native Japanese squirrels are not distributed in the area where Pallas's squirrel is known to be established, and therefore both squirrels are currently isolated each other (Miyamoto et al., 2004). However, Pallas's squirrel is gradually spreading through green areas (Takahata et al., 2020), and it is still possible that the exotic louse will be transported to mountainous areas where the native squirrel lives.

## 5 Conclusions

The present study suggests that earlier studies have underestimated the diversity and abundance of ectoparasites associated with invasive Pallas's squirrel in Kanto region of Japan and that parasite spillover and spillback risks need to be adjusted upwards. However, the parasitic situation in other regions of Japan where Pallas's squirrel has already been established or its invasion is newly recognized remains still unknown. Particularly in western Japan, where vector-borne diseases are more pronounced (Yamaji et al., 2018; Kinoshita et al., 2021), both the spillover and spillback risks of ectoparasites via this invasive squirrel may have to be taken more seriously. The effort invested in eradication and/or control measures should vary depending on whether a parasite-free situation occurs in these areas, or whether ectoparasites are infecting this invasive squirrel and enhancing the risks to other organisms including humans. Since existing host-parasite relationships can be easily modified by small exotic mammal invasions as is the cases in middle- and large-sized species (e.g. Doi et al., 2020, 2021), it is necessary to understand the spatiotemporal variability of ectoparasite distribution and abundance for optimal management planning, with adopting the fur shaving as applied in this study and other comprehensive collecting methods.

## Ethical statement

The present study was conducted in accordance with the guidelines for the care and use of laboratory animals at Azabu University, and was approved by the ethics committee (No. 190701–3).

## Funding

Not applicable.

## Author's contributions

All authors contributed to the study conceptiong and design. Material preparation, data collection, and analysis were performed by YE, SH, YO, AB, and YO. Project administration, interpretation of data, and revision the manuscript critically for important intellectual content were conducted by TS. The first draft of the manuscript was written by HK and YE. All authors commented on previous versions of the manuscript, and read and approved the final manuscript.

## Declaration of competing interest

On behalf of all authors, the corresponding author states that there is no conflict of interest.
